# Hearing Tones, Missing Boundaries: Cross-Level Selective Transfer of Prosodic Boundaries Among Chinese–English Learners

**DOI:** 10.3390/bs15121605

**Published:** 2025-11-21

**Authors:** Lan Fang, Zilong Li, Keke Yu, John W. Schwieter, Ruiming Wang

**Affiliations:** 1 English Department, School of Foreign Studies, Guangzhou University, Guangzhou 510006, China; robinfl@163.com; 2 Guangdong Key Laboratory of Mental Health and Cognitive Science, Key Laboratory of Brain, Cognition and Education Sciences, Ministry of Education, and Center for Studies of Psychological Application, School of Psychology, South China Normal University, Guangzhou 510631, China; lizilong0000@outlook.com (Z.L.); kkyu@m.scnu.edu.cn (K.Y.); 3 Language Acquisition, Multilingualism, and Cognition Laboratory/Bilingualism Matters, Wilfrid Laurier University, Waterloo, ON N2L 3C5, Canada; 4 Department of Linguistics and Languages, McMaster University, Hamilton, ON L8S 4L8, Canada; 5 School of Education, University College Dublin, D04 C1P1 Dublin, Ireland

**Keywords:** perception, prosodic boundaries, second language learning, cue-weighting transfer hypothesis

## Abstract

Second language (L2) learners often struggle to process prosodic boundaries, which are essential for speech comprehension. This study investigated the nature of these difficulties and how first language (L1) cue-weighting strategies transfer to L2 processing among Chinese (Mandarin)–English learners. The rising pitch that cues English phrase boundaries acoustically overlaps with functionally distinct Chinese lexical tones. Through two experiments comparing Chinese–English learners and native English speakers, we assessed sensitivity across lexical constituent, phrase, and sentence boundaries and manipulated acoustic cues (pause, lengthening, pitch) to estimate their perceptual weights during phrase-boundary identification. L2 learners showed reduced discrimination sensitivity only at the phrase level, performing comparably to native speakers at lexical constituent and sentence boundaries. For phrase boundaries, learners over-relied on pitch and under-relied on pre-boundary lengthening compared to native speakers, though both groups weighted pauses strongly. This selective deficit implicates the transfer of L1 cue-weighting strategies more than a global knowledge deficit. Our findings support a dynamic transfer model where L1 sensitivity to lexical tone transfer of L2 phrase perception, elevating the weight of pitch. While learners show partial adaptation, these results refine the Cue-Weighting Transfer Hypothesis by demonstrating that L2 prosodic acquisition involves both integrated L1 transfer and L2-driven reweighting strategies.

## 1. Introduction

Prosodic boundaries are places in language in which breaks are marked by acoustic cues (e.g., pauses, pitch changes, etc.). These boundaries often coincide with grammatical boundaries and play a crucial role in language comprehension by helping individuals to more efficiently parse target structures. Prosodic boundaries serve as a framework that segments speech flow into chunks such as words, phrases, and clauses (Frazier et al., 2006), forming the foundation of the highly structured language system. They manifest syntactic, semantic, or pragmatic rules, thereby disambiguating the auditory sentences (Nickels et al., 2013). Prosodic boundaries also assist listeners in decomposing continuous speech signals and extracting meaningful units for language acquisition (Holzgrefe-Lang et al., 2016), while facilitating fluency in reading comprehension (Holliman et al., 2017).

However, prosodic boundaries can pose challenges for L2 learners. For instance, studies have shown that L2 learners’ performance on recall tasks (Pennington & Ellis, 2000; Schmidt et al., 2020) and naturalness judgment tasks (Nickels & Steinhauer, 2016) using L2 prosodic boundaries is lower than in their native language or when compared to native speakers. The causes of this challenge have been explained by divergent views. Some researchers attribute it to L2 learners’ lack of awareness of prosodic boundaries (Pennington & Ellis, 2000). Other researchers suggest that there are fundamental differences in the processing mechanisms between L2 learners and native speakers (Schmidt et al., 2020), or that the impact of weighting strategies transfers from their first language (L1) (Baek, 2022).

To elucidate the nature and mechanisms of these difficulties, we conducted two experiments among groups of native English speakers and Chinese learners of English. We focus on speakers of these two languages for two reasons. First, English and Chinese have divergent acoustic cue realizations for prosodic boundaries beyond chunk boundaries, and our study expands prior studies (e.g., Schmidt et al., 2020) by examining more than one boundary type. Second, cross-linguistic differences in intonational phrase marking place Chinese learners in a theoretically informative position not addressed in previous work, yielding conflicting predictions under the Cue-Weighting Transfer Hypothesis. Examining how these learners process English prosodic boundaries therefore offers the potential to refine accounts of transfer mechanisms.

### 1.1. Challenges of Prosodic Boundaries in L2s

L2 learners face difficulties in utilizing prosodic boundaries, often failing to achieve comparable efficiency or accuracy as native speakers. For example, in a recognition memory task, Pennington and Ellis (2000) found that highly proficient Cantonese–English learners struggled to recognize sentences in which word sequences were identical but the prosodic boundaries differed, such as “The fight is over Fred” vs. “The fight is over, Fred.” In a second experiment in which the participants were explicitly instructed to attend to prosodic differences, their task performance did not improve. The authors attributed these findings to the absence of teaching pedagogies that focus on phonological instruction in the learners’ educational background. However, the study did not provide evidence demonstrating that L2 learners indeed lacked knowledge of prosodic boundaries. In another study, Baek (2022) showed that Korean–English leaners’ performance on an ambiguous sentence comprehension task was modulated by their L2 age of acquisition and L2 proficiency level. The author argued that L2 learners’ processing of prosodic boundaries is limited by cognitive constraints, particularly lower L2 processing capacity.

Similarly, in the aforementioned digit recall task conducted by Schmidt et al. (2020), a group of native English speakers and a group of highly proficient Greek–English learners performed the task in both their languages under conditions with and without prosodic boundaries. The results showed that the presence of prosodic boundaries facilitated recall for both groups, but only in their L1s. The authors attributed these findings to the notion that because prosody is a deep structure, the participants were unable to process prosodic boundaries in their L2 (i.e., non-automatic processing). While this interpretation is plausible, it appears to overlook the phonological differences in prosodic boundaries between the two languages. Specifically, at intonational phrase prosodic boundaries, there is a falling tone in Greek, but a raising tone in English. It is possible that these typological differences may account for some of their findings. Indeed, findings from a neurolinguistic study also indicated that during naturalness judgment tasks involving prosodic boundaries in ambiguous sentences, the performance gap between highly proficient German–English learners and native English speakers was very small, whereas the gap for Chinese learners was significantly larger (Nickels & Steinhauer, 2016). This pattern suggests that the degree of typological difference between languages may be a factor contributing to learners’ challenges in utilizing prosodic boundaries in their L2s.

In sum, behavioral and EEG evidence shows that L2 learners often struggle to process prosodic boundaries. Because prior studies rarely have verified learners’ prosodic knowledge, often focusing on a single boundary type (e.g., intonational phrases), the source of these difficulties remains unknown. They may reflect gaps in prosodic knowledge, qualitative differences in L1 vs. L2 processing mechanisms, or transfer of L1 cue-weighting strategies. To examine these possibilities, we designed a novel task in which we manipulate boundary type to independently assess prosodic knowledge and online processing.

### 1.2. The Cue-Weighting Transfer Hypothesis

The Cue-Weighting Transfer Hypothesis in L2 speech processing claims that L1 cue weights transfer to L2 processing, with two caveats: (i) cues that are highly weighted in the L1 tend to retain their weight in the L2, even when their functional role or positional realization differs; (ii) when multiple cues are available in the L2 and learners’ relative cue hierarchy is shaped by their L1. Views based on cue-weighting theory suggest that language acquisition involves learning the distribution of weighting patterns assigned to acoustic cues within the target language (Escudero et al., 2009; Kluender et al., 1998). For late L2 learners, the weighting patterns of acoustic cues from their L1 become entrenched before they begin to learn their L2. It is possible that these L1-acquired cue-weighting patterns may transfer to processing strategies that learners employ in their L2. This notion has been elaborated on in the Cue-Weighting Transfer Hypothesis (Chang, 2018; Baek, 2022; Chrabaszcz et al., 2014; Qin et al., 2017; Schertz et al., 2015; Tremblay et al., 2018; Y. Zhang & Francis, 2010). The hypothesis posits that cues that are more heavily weighted in an L1 (i.e., those which have greater assigned importance) are more likely to be utilized in L2 processing (e.g., Baek, 2022; Francis & Nusbaum, 2002; Holt & Lotto, 2006; Iverson et al., 2003; McAllister et al., 2002).

L1 acoustic cues can confer advantages in L2 tasks, even when the cues are not functionally equivalent across languages (Lin et al., 2014; Qin et al., 2017). Interestingly, these transferred cues need not occur in phonologically equivalent positions in the two languages. For example, in the study by Lin et al., Korean–English learners had poorer performance compared to Chinese–English learners on a series of English word judgment tasks. The authors attributed these effects to the absence of lexical tone in Korean.

When two or more cues are present, the weighting hierarchy of the cues is also influenced by the L1. For instance, Tremblay et al. (2018) investigated how the functional weighting of stress cues in an L1 predicted L2 speech segmentation. When comparing English–French and Dutch–French learners to native French speakers, the researchers found that Dutch participants outperformed English speakers and even surpassed native French speakers. The authors argued that because lexical stress is a primary cue for word segmentation in Dutch, but is secondary in English, these findings offer further support for the transfer of weighting strategies.

Another example of such transfer comes from a study on L2 learners’ perception of boundaries in ambiguous sentences. Baek (2022) employed a forced-choice task to investigate how native English speakers and Korean–English learners utilized pause, duration, pitch, and intensity cues in boundary words to resolve syntactic ambiguity in English (specifically high vs. low attachment). The results revealed that native English speakers prioritized pitch cues, followed by pause and intensity cues. Due to the differing weighting strategies between Korean and English, the Korean learners exclusively relied on pause cues for processing English boundaries. In a study by Lin et al. (2014), Korean learners of English performed worse than Chinese learners on English lexical judgment tasks, consistent with the absence of lexical tone in Korean and its presence in Chinese (Lin et al., 2014). Moreover, Kim (2020) showed that speakers of dialects of Korean with lexical–pragmatic tonal accents showed better perception of English lexical stress than speakers of dialects without such accents.

### 1.3. Criticisms of the Cue-Weighting Transfer Hypothesis

Predictions from the Cue-Weighting Transfer Hypothesis regarding the perception of prosodic boundaries among Chinese–English learners have yielded contradictory results. On the one hand, intonational phrase boundaries in Chinese do not include rising tone (which means a zero-weighting for pitch movement), whereas many specific English intonational phrase boundaries (e.g., listing) often involve a half-rising tone (i.e., continuation rise) (Pu, 2003, p. 117, also see the description of acoustic correlates of English chunks by Schmidt et al., 2020). Given that pitch movement carries zero weighting in marking Chinese prosodic phrase boundaries, according to the Cue-Weighting Transfer Hypothesis, L1 Chinese speakers should not exhibit reliance on this cue when processing English phrase boundaries. This predicted reliance is significantly weaker than the weight assigned to rising pitch in English. On the other hand, pitch movement carries a significantly higher functional load in Chinese than in English, as tones are crucial in distinguishing meanings. The most classic example is the syllable /ma/, which can mean “mother” (妈, mā), “hemp” (麻, má), “horse” (马, mǎ), or “scold” (骂, mà), depending on its tone (Chao, 1968; Duanmu, 2007). Consequently, pitch movement is believed to play a significant role in L2 processing (Qin et al., 2017). When considering these two differing predictions, a paradox clearly arises.

Taken together, these factors underscore the uniqueness of Chinese learners’ processing of English intonational phrase boundaries compared to most studies of prosodic transfer. The resulting complexity differs both from transfer patterns reported for Chinese- or Korean-speaking learners of English stress and from phenomena observed among Korean listeners who disambiguate ambiguous English boundaries. Accordingly, our aim is to examine Chinese speakers’ processing of English prosodic boundaries, an area of inquiry which merits dedicated, in-depth investigation.

## 2. Present Study^1^

In the context of prior work, there are two important issues that have not yet been explored. First, previous research has not examined the effects of transfer when a cue has functionally conflicting roles in the two languages, e.g., having zero weight in some contexts but high weight in others. Second, when multiple L1 cues transfer concurrently, it is unclear whether their relative ordering is reconfigured and if so, how such reordering interacts with the L2’s cue-weighting system. In the present study, we address these issues. We conducted two experiments to test whether Chinese–English learners and English native speakers differentially weight pitch, pause, and pre-boundary-lengthening across boundary types, and whether explicit prosodic knowledge predicts their use of cues.

Experiment 1 tested whether difficulties arise from insufficient knowledge of prosodic boundaries or from global processing constraints. If a knowledge deficit is the challenge, L2 learners should underperform monolinguals across boundary types. However, if processing constraints dominate, group differences should be limited to specific boundary types. Experiment 2 examined boundary-cue transfer and lexical-tone transfer. Regarding boundary-cue transfer, the Cue-Weighting Transfer Hypothesis predicts reduced reliance on pitch among Chinese–English learners relative to monolinguals English speakers when identifying English phrase boundaries. For lexical-tone transfer, the high functional load of pitch in Chinese predicts greater reliance on pitch by learners than by monolinguals. Because duration (pre-boundary lengthening) serves as a weaker pragmatic cue than pitch in the learners’ L1 Chinese, we also test whether these individuals weigh duration less than pitch during L2 boundary processing.

### 2.1. Data Analyses

The data in the two experiments were analyzed using repeated measures ANOVA in SPSS 27.0. Our analyses are informed by Signal Detection Theory (SDT). SDT is an important analytical framework in perception, cognition, and decision-making research (Green & Swets, 1966; Lynn & Barrett, 2014) that is used to examine how different factors influence information discrimination (Gawronski et al., 2023). By analyzing the different outcomes of information discrimination (hits, misses, false alarms, and correct rejections), we can gain a deeper understanding of the various factors that affect misinformation discrimination.

SDT distinguishes between two important factors influencing individual judgments (Green & Swets, 1966; Lynn & Barrett, 2014): discriminability (d’) and decision criterion (C). Discriminability reflects an individual’s ability to distinguish between true and false information. The decision criterion represents an individual’s tendency to classify information as true or false when making judgments. After converting the hit and false alarm rates into z-scores from the standard normal distribution, discriminability was calculated using the formula d’ = z(H) − z(FA), and decision criterion was calculated as C = −0.5 × [z(H) + z(FA)]. By using these formulas, we can quantify participants’ ability to differentiate between prosodic boundary types, independent of their response biases.

### 2.2. Experiment 1

In Experiment 1, we examine the perception of English prosodic boundaries using a 2 (type: English native speakers vs. L2 learners) × 3 (prosodic level: no boundary vs. prosodic phrase vs. sentence) mixed design with identification accuracy as the dependent variable.

#### 2.2.1. Participants

According to a G*Power 3.1.9.2 analysis (a partial eta squared [*η_p_*^2^] value of 0.025, effect size f = 0.23, an alpha (α) threshold of 0.05, and 0.95 power, the minimum sample size was 84 (42 per group). As such, we recruited 42 Chinese–English learners and 50 English monolinguals. Because three participants in the former group failed to complete the task and one participant from the latter group exceeded the time limit in the experiment, the final sample included 39 Chinese–English learners and 49 English monolinguals.

The monolinguals, aged 18 to 32 (M = 21.87, SD = 3.56; M = 5), were university students in the United States. The Chinese–English learners were fourth-year undergraduate students (M = 22.08, SD = 0.48; Male = 6) majoring in English at Guangzhou University in South China. They began formal, classroom-based English instruction around the age of six. By their fourth year of university, their English proficiency was estimated to be at the C1–C2 level of the common European framework of reference (CEFR) for languages with the reference to New English Curriculum distributed by the Chinese Ministry of Education (2018). The participation criteria for the learners were as follows: (1) Native Chinese speakers of standard Chinese; (2) English learning experience primarily through classroom instruction; (3) No more than 6 months of stay in an English-speaking country before the experiment.

All participants were renumerated for their time in accordance with local standards. Research ethics approval was granted by the same institution, and all participants provided their informed consent prior to taking part in the study.

#### 2.2.2. Materials^2^

Compound words (e.g., jellybean) were used to prepare the experimental materials. First, sentences were formed based on the characteristics of compound words as follows:

1. I bought jelly#beans and cookies as presents for him. (lexical constituent boundary)
2. I bought jelly#, beans, and cookies as presents for him. (intonational phrase boundary)
3. I bought jelly#. Beans and cookies were presents for him. (sentence boundary)

These sentences were then read aloud by a 25-year-old male native English speaker from the United States. The recordings were made in a professional studio using specialized equipment and software (cooleditor) with a sampling rate of 44,100 Hz, stereo, 16-bit. To prevent the reader from unintentionally exaggerating differences between prosodic levels due to contrast, the reading list included 20% filler sentences in a pseudo-random manner. Additionally, there was a gap of at least ten sentences between the three sentences with the same word order.

Subsequently, Propraat was used to extract the duration of pauses, pitch slope, and duration of lengthening on Praat (X. T. Zhang, 2012). For example, in the speech fragment //chocolate, yogurt, and candy//, the pause was measured as the length of the silent period between “chocolate” and “yogurt.” Pitch was calculated as the difference between the starting and ending pitch (in Hz) of the last syllable of “chocolate” divided by the time interval between them, using the formula (Tonset − Toffset)/duration. A positive value indicates a rising pitch contour, while a negative value represents a falling pitch contour. (Pre-boundary) lengthening referred to the length of the syllable /lɪt/.

A one-way ANOVA was used to analyze the three parameters to compare the phonetic differences in prosodic boundaries at different levels. The results showed significant differences in delay cues before the boundary for the three prosodic levels, *F*(2,359) = 66.93, *p* < 0.001, with the shortest lengthening duration at lexical constituent boundaries (e.g., /i/ of y in jelly#bean), the longest at phrase boundaries, and the lengthening before sentence boundaries falling in between. There were also significant differences in pause length (i.e., the length of the silent period), *F*(2,359) = 941.3, *p* < 0.001, with the shortest pauses at lexical constituent boundaries (with no pauses present), the longest pauses at sentence boundaries, and pauses at phrase boundaries in between. There were significant differences in pitch slope among the three prosodic levels, *F*(2,359) = 54.1, *p* < 0.001. At lexical constituent boundaries, there was a slight pitch movement from low to high (positive slope, *M* = 24.03). Pitch movement was greatest at phrase boundaries, showing a rising pitch contour (*M* = 107.42). At sentence boundaries, pitch movement was in the opposite direction than at intonational phrase boundaries, showing a falling pitch contour (negative slope, *M* = −43.84).

Preparation of experimental stimuli involved cutting the speech fragments from the beginning of the sentence to just before the onset of the second word. For example, the spoken sentence “I bought chocolate yogurt, and candy as presents for him” was cut to form the experimental stimulus “I bought chocolate.” A total of 24 sets of sentences were prepared, resulting in 72 (i.e., 24 × 3) stimuli. Additionally, based on the types of target sentences, filler sentences were created (e.g., “She is introducing local foods and historical sites to her friends”), with a target-to-filler sentence ratio of 2:1. The stimuli were presented in a pseudo-randomized order, using a balanced Latin square for inter-item presentation.

#### 2.2.3. Procedure

The native English speakers completed the task using the online experimental platform Prolific (prolific.com) due to the COVID-19 pandemic. They were required to complete the experimental task alone in a quiet environment, using a computer and headphones. L2 learners completed the experiment in a behavioral laboratory. The experimental procedure was programmed and run using E-prime 3.0. Participants sat approximately 50 cm in front of a 21-inch Lenovo-M800EE computer monitor, wearing LE5 (LingJi-le5) headphones.

**Figure 1:**
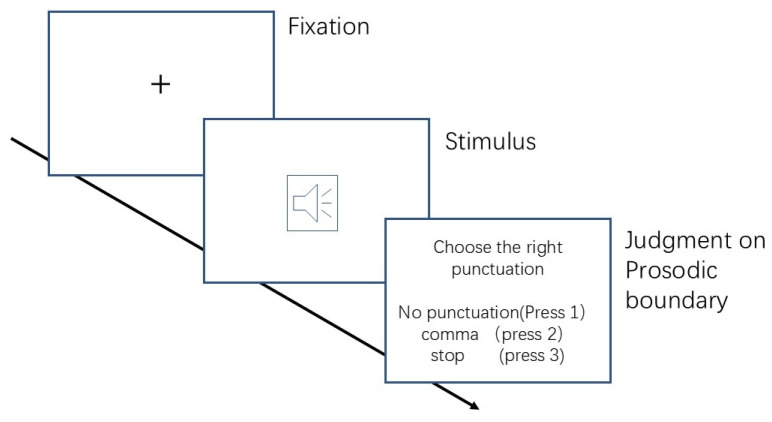
Procedure of Experiment 1.

As shown in Figure 1, a fixation point was first displayed on the screen for 500 milliseconds. Then, a speech fragment was played through the headphones, followed by the task instructions (“Please choose the appropriate punctuation mark for the phrase you have heard”). At the same time, three options (1. No punctuation; 2. Comma; 3. Period) were presented on the screen in 36-point Song typeface. Participants had five seconds to respond, and if they did not respond within this time, the next trial automatically proceeded. Before the start of the experiment, participants had a brief practice session that included feedback. The entire experiment lasted approximately 30 min.

#### 2.2.4. Results

**Figure 2:**
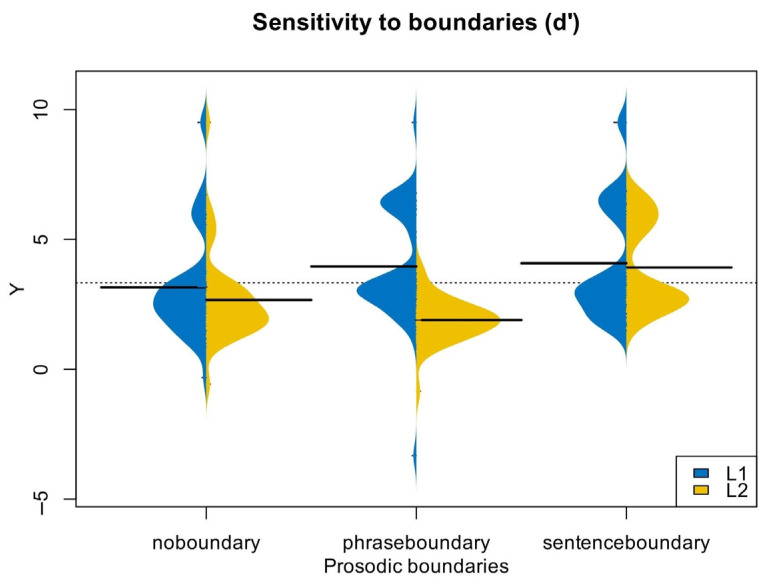
Sensitivity to prosodic boundaries (*d*’) in Experiment 1.

The results showed that all participants had sensitivity to boundary types greater than at random (d’ > 0), *t*(276) = 26.94, *p* < 0.001. Both English native speakers (*M* = 4.09 ± 2.24) and Chinese–English learners (*M* = 3.92 ± 1.70) showed high sensitivity to sentence boundaries. However, the two groups differed in their sensitivity to the other two levels of prosodic boundaries: the native speakers had the lowest sensitivity to lexical constituent boundaries (*M* = 3.16 ± 2.05), while the L2 learners had the lowest sensitivity to intonational phrase boundaries (*M* = 2.67 ± 1.73). Table 1 and Figure 2 illustrate these results.

The results of the ANOVA showed a significant main effect of prosodic boundary type, *F*(2,172) = 18.27, *p* < 0.001, *η_p_*^2^ = 0.175. The interaction between sensitivity and participant type was also significant, *F*(2,172) = 22.43, *p* < 0.001, *η_p_*^2^ = 0.123. Further simple effects tests (after Bonferroni correction) revealed a significant effect of participant type and phrase boundaries, *F* = 31.86, *p* < 0.001, but no significant effect of lexical constituent boundaries, *F*(2,172) = 1.40, *p* = 0.24, or sentence boundaries, *F*(2,172) = 0.14, *p* = 0.71. These findings indicate that the two groups significantly differed in their sensitivity to intonational phrase boundaries, but not to the other two levels. There were significant differences in prosodic boundary types for both participant groups (*p*s < 0.002). Native speakers’ sensitivity increased with prosodic boundary level, whereas L2 learners had the highest sensitivity to sentence boundaries and the lowest to phrase boundaries, which was inconsistent with the break index of the English prosodic hierarchy (Price et al., 1991). These results suggest that Chinese–English learners’ ability to recognize lexical constituent boundaries and sentence boundaries is comparable to that of English native speakers, but their ability to recognize phrase boundaries is significantly lower.

**Figure 3:**
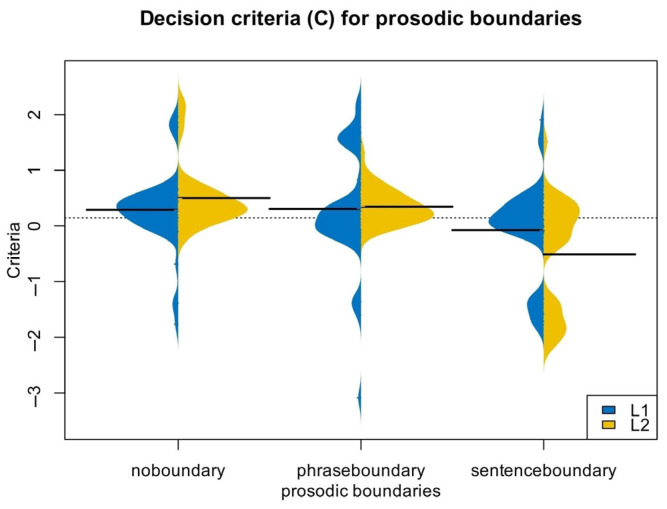
Decision Criteria (*C*) for prosodic boundaries in Experiment 1.

The ANOVA results for *C* showed a significant main effect of prosodic level, *F*(2,172) = 15.72, *p* < 0.001, *η_p_*^2^ = 0.16. There was also a significant interaction between prosodic level and participant type, *F*(2,172) = 3.09, *p* = 0.048, *η_p_*^2^ = 0.035. Further simple effect tests revealed a significant effect of participant type on sentence boundaries, *F*(2,172) = 4.94, *p* = 0.029, but no significant effect on lexical constituent boundaries, *F*(2,172) = 2.42, *p* = 0.123, or intonational phrase boundaries, *F*(2,172) = 0.06, *p* = 0.81. Additionally, no significant differences in boundary types emerged among the monolingual group, *F*(2,172) = 2.86, *p* = 0.06, unlike for L2 learners who exhibited significant differences, *F*(2,172) = 14.61, *p* < 0.001. These results indicate that Chinese–English learners used different criteria in distinguishing prosodic levels compared to English monolinguals, particularly for sentence boundaries. Specifically, L2 learners were significantly more accurate in identifying sentence boundaries than monolinguals. This may be because L2 learners are more cautious in recognizing acoustic cues of word and phrase boundaries—and potentially more so for this particular sample whose L1 is a tonal language—but are less sensitive to phrase boundaries. As a result, Chinese–English learners tend to categorize items that do not belong to a sentence as sentence boundaries, leading to a notably low *C*-value for sentence boundaries. These findings are shown in Table 2 and Figure 3.

In sum, L2 learners faced challenges in differentiating English prosodic levels, as reflected by their low sensitivity (*d’*) to intonational phrase boundaries. In Experiment 2, we explore the underlying mechanisms behind this finding.

### 2.3. Experiment 2

In Experiment 2, we examine the extent to which individuals rely on different acoustic cues by investigating the weights they assign to various cues during intonational phrase boundary processing. The experiment employs a 2 (type: native English speakers vs. L2 learners) × 4 (single-cue conditions: pause vs. lengthening vs. pitch vs. baseline) mixed design, in which the single-cue condition is a within-subjects variable, and identification accuracy is the dependent variable. Our analyses in Experiment 2 only include a repeated-measures ANOVA; we did not apply the SDT framework as was the case in Experiment 1.

#### 2.3.1. Participants

Participants were recruited using the same method as in Experiment 1. None of the individuals who participated in Experiment 1 took part in Experiment 2. A G*power analysis (a partial eta squared [*η_p_*^2^] value of 0.05, effect size *η*^2^ = 0.23, an alpha (α) threshold of 0.05, and 0.80 power) showed that the required sample size was 54 (27 for each group). Accordingly, we recruited 30 English monolinguals and 30 Chinese–English learners. Because three monolinguals and one L2 learner failed to complete the experiment, the final sample included 27 monolinguals and 29 L2 learners.

#### 2.3.2. Materials

Forty-eight sentences (e.g., “I saw tea, cups, and spoons on the table.”) containing prosodic phrase boundaries were selected from the materials of Experiment 1 to serve as target sentences in Experiment 2. Additionally, 48 sentences containing lexical constituent boundaries (e.g., “I saw sea#birds and seals on the boat”) were selected as filler sentences. No target sentences or filler sentences were from the same set. Target sentences were then manipulated to serve as the following conditions: lengthening-only, pitch-only, pause-only, and baseline following the paradigm in Petrone et al. (2017). Baseline sentences were not modified and, thus, included their original acoustic cues. After manipulating the acoustic cues in the stimuli conditions, a total of 48 × 4 target sentences were obtained. These sentences were balanced according to a Latin square design.

#### 2.3.3. Procedure

The experiment was presented to English native speakers on the Prolific platform. The participants were asked to sit 50 cm in front of a computer screen, wear headphones, and complete the experimental tasks in a quiet environment. The L2 learners completed the experiment in a behavioral lab. E-prime 3.0 was used to run the experiment.

**Figure 4:**
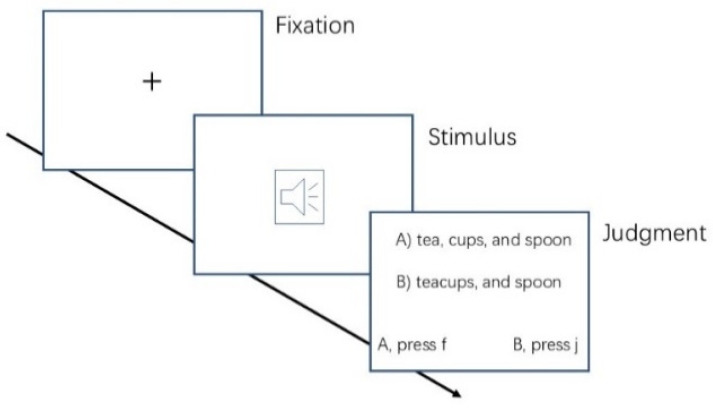
Procedure of Experiment 2.

The procedure for both groups was as follows. First, instructions appeared on the screen. After confirming their understanding of the instructions, participants went through a practice set (without feedback) before the formal experiment began. A fixation cross was first displayed on the screen for 500 milliseconds (see Figure 4). Subsequently, an auditory stimulus was played through the headphones, followed by two options (e.g., (A) tea, cups, and spoons; (B) teacups and spoons) displayed on the screen in Times New Roman font, representing phrase boundaries (targets) and word boundaries (fillers). Participants used the keyboard to complete a judgment task based on which sentence fragment on the screen matched the one they heard. There was a break after every 30 sentences, with the duration of the break being determined by each participant. The entire experiment lasted approximately 35 min.

#### 2.3.4. Results

**Figure 5:**
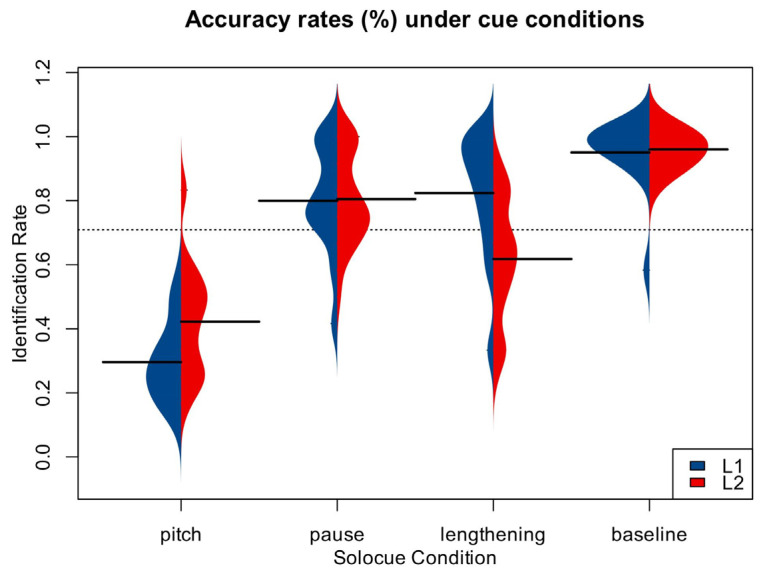
Identification rates (%) under cue conditions in Experiment 2.

Given that a lack of prosodic boundary awareness affects speech comprehension (Y. Zhang & Ding, 2020), we used identification rates of baseline judgments as a standard. We considered accuracy below 80% to imply a lack of prosodic boundary awareness and therefore excluded these data from the analyses. The identification rates for baseline and cue conditions can be seen in Table 3 and are illustrated in Figure 5.

The results of the analyses showed a significant main effect of acoustic cues, *F*(3,162) = 229.45, *p* < 0.001, *η_p_*^2^ = 0.81. There was also a significant interaction between acoustic cues and participant type, *F*(3,162) = 17.20, *p* < 0.001. Further simple effects analyses revealed that participant type affected accuracy rates of pitch, *F*(3,162) = 9.58, *p* = 0.003, and lengthening, *F*(3,162) = 16.30, *p* < 0.001, but not of baseline or pause levels. That is, there was a significant difference between L2 learners and native speakers in their use of pitch and delay cues, but not in their use of pause cues. The results also showed that acoustic cues had a significant effect on both participant groups, *F*(3,162) = 144.39, *p* < 0.001; *F*(3,162) = 100.71, *p* < 0.001. To further understand the weighting sequence of individual cues, we conducted one-way ANOVAs on the accuracy rates for each group across the four conditions. The results revealed a significant main effect of acoustic cues for monolinguals, *F*(3,162) = 96.10, *p* < 0.001. Post hoc analyses indicated no significant difference between pauses and lengthening but demonstrated significant differences between pitch and baseline (*p*s < 0.05). For L2 learners, the main effect of acoustic cues was also significant, *F*(3,162) = 70.91, *p* < 0.001, with post hoc tests showing significant differences between baseline, pauses, lengthening, and pitch (*p*s < 0.01).

Finally, to compare awareness of prosodic phrase boundaries and perceptual skills between the two groups, we analyzed the differences in accuracy rates under baseline conditions. These accuracy rates were above 95% for both groups, and there were no significant differences between the two, *t*(54) = 0.404, *p* = 0.688. This suggests that both monolinguals and L2 learners had metacognitive awareness and perceptual skills for recognizing prosodic phrase boundaries (Y. Zhang & Ding, 2020). Therefore, we attribute the differences between English monolinguals and Chinese–English learners to the influence of their L1 backgrounds.

The accuracy rates under single-cue conditions were used to determine the weight assigned to each cue during phrase boundary identification. If a cue is given a higher weight, we can infer that it plays a more decisive role in an individual’s identification process. In other words, the individual is more likely to identify the stimulus as a phrase boundary, relying more on that particular cue. Based on the accuracy rates, the weighting sequence of the cues for the monolingual group was as follows: pause (*M* = 79.94%, *SD* = 16.87%) = lengthening (*M* = 82.41%, *SD* = 19.25%) > pitch (*M* = 29.63%, *SD* = 12.52%). For the L2 learner group, the weighting sequence from highest to lowest was as follows: pause (M = 80.46%, *SD* = 14.13) > lengthening (*M* = 61.78%, SD = 18.97%) > pitch (*M* = 42.24%, *SD* = 17.39%). These patterns indicate a significant difference in acoustic cue-weighting strategies between the two groups, particularly for lengthening and pitch. Specifically, monolinguals relied more on lengthening and less on pitch than L2 learners. This suggests that for L2 learners, the pitch weighting strategy from the L1 lexical level influenced their processing of L2 prosodic phrase boundaries.

## 3. Discussion

### 3.1. The Selective Difficulty of Perceiving Prosodic Boundaries

Some theoretical accounts propose that L2 learners either lack awareness of target-language prosodic boundaries (Pennington & Ellis, 2000) or fail to process them as deeply as native speakers (Schmidt et al., 2020). Our results challenge both of these accounts. We argue that the observed difficulties are neither a consequence of lacking boundary knowledge nor evidence of a global processing deficit. Instead, our findings revealed that they are boundary specific. In Experiment 1, L2 learners’ accuracy was on par with native speakers in identifying both lexical constituent boundaries and sentence boundaries yet showed significantly lower sensitivity (d′) to prosodic phrase boundaries. This pattern indicates a selective locus of difficulty at the prosodic-phrase level rather than a general impairment in prosodic processing.

This discrepancy likely reflects cross-language differences in the cue composition of intonational phrase boundaries. In English, such boundaries are typically signaled by several cues—pauses, pre-boundary lengthening, and pitch accenting or F0 contouring (Price et al., 1991; Wightman et al., 1992). In Chinese, boundaries are mainly marked by pauses, pre-boundary lengthening, and pitch reset (X. Yang et al., 2014; X. T. Zhang, 2012). Because these cues diverge, L1-conditioned cue weights can interfere with L2 prosodic parsing (Kim, 2020; Tremblay et al., 2018).

Our findings therefore support a more nuanced account: L2 difficulties with prosodic boundaries arise not from lack of awareness or a global processing deficit, but from the transfer of cue-weighting strategies from the L1 to the L2, consistent with work by Baek (2022). This transfer can impede learners’ uptake of task-critical cues in the target language, especially at prosodic levels where cue configurations diverge from those of the L1. We will next discuss how L1 cue weights are projected onto L2 processing and how this mechanism explains the selective difficulty observed for intonational phrase boundaries.

### 3.2. The Transfer of Cue-Weighting Strategies from L1 to L2

Our results indicate that L2 learners and native speakers do not differ in their explicit awareness or overall sensitivity to intonational phrase boundaries, but they diverge in how they assign weight to the underlying acoustic cues. In Experiment 2’s baseline condition, both groups achieved ≥ 95% accuracy, corroborating the notion that learners do not lack perceptual ability for these boundaries (Y. Zhang & Ding, 2020). Under single-cue conditions, accuracy declined markedly for both groups, consistent with the view that boundary perception is an integrative, multi-cue computation rather than a single-cue detection (Price et al., 1991). This underscores the value of examining speech units defined by partially redundant cues—contexts in which no single feature suffices to identify the category—rather than the single-feature cases that have been examined in prior work (e.g., Dupoux et al., 2008). Below we elaborate on how L1-conditioned cue weights transfer to the L2 and how such transfer manifests.

Our findings showed that when processing English intonational phrase boundaries, L2 learners did not transfer isolated cues, but instead, they projected a partially integrated L1 cue-weighting schema into their L2. This is evident in the distinct weighting hierarchies that were observed across cues. For monolinguals, the estimated weights were as follows: pause (M = 79.94%) = lengthening (M = 82.41%) > pitch (M = 29.63%). For L2 learners, they were as follows: pause (M = 80.46%) > lengthening (M = 61.78%) > pitch (M = 42.24%). Thus, compared to monolinguals, L2 learners up-weighted pitch and down-weighted lengthening, while maintaining a strong reliance on pause. These findings reflect a composite mechanism in which L1 transfer is coupled with adaptive reweighting to L2 cue diagnostics.

With respect to the sources and consequences of this reweighting, first, regarding the pause cue, both groups exhibited recognition rates exceeding 80%, with no significant differences between the groups. This supports the view that pause serves as a cross-linguistic “meta-acoustic cue”, one which has a strong physiological basis and is a universally employed prosodic strategy across human languages (Holzgrefe-Lang et al., 2016). It also plays a dominant role in language acquisition in infants (Seidl & Cristià, 2008). Its effectiveness as a boundary marker is fully utilized in both L1 and L2 acquisition, resulting in the successful and complete transfer of its high weighting from L1 to L2.

Second, for the lengthening cue, L2 learners demonstrated significantly lower weighting compared to monolinguals, reflecting negative transfer from L1 to L2 (Nenonen et al., 2003). Although lengthening is also used to mark prosodic boundaries in Chinese, its functional load is considerably lower than in English. In Chinese, lengthening is primarily associated with pragmatic functions (Y. F. Yang, 1997), whereas in English, it serves as a crucial grammatical and semantic cue, sometimes even comparable to pause in importance (Aasland & Baum, 2003). This difference in functional load leads to lower efficiency among L2 learners when processing lengthening in English.

Notably, despite the presence of negative transfer, L2 learners’ weighting of lengthening (61.78%) was higher than that of native Chinese speakers in other studies (<50% in X. Yang et al., 2014). This indicates that L2 learners actively adjust their cue weighting based on the functional demands of the target language. When Chinese–English learners engage in English prosodic phrase processing, they recognize lengthening as a highly reliable and functionally significant marker in English. Their cognitive systems become aware of this and proactively increase the weighting of this cue to better adapt to the requirements of English. These adjustments represent successful adaptive transfer, elevating their weighting of lengthening from a low level in the L1 (<50%) to a medium-high level in the L2 (61.78%). Although it has not yet reached native-like levels, this constitutes significant progress.

L2 learners assigned greater weight to pitch than monolinguals. At first glance, this conflicts with the Cue-Weighting Transfer Hypothesis which holds that cues with near-zero L1 weight should remain weakly weighted in the L2. We interpret this pattern as cross-unit transfer of L1 pitch sensitivity. In Chinese, pitch contributes little to prosodic-phrase boundary marking, yet it carries a high lexical functional load. This entrenched sensitivity generalizes to L2 boundary perception despite the functional shift, allowing learners to detect subtle F0 variations. Consistent with this account, learners outperformed monolinguals in pitch-only trials.

At the same time, learners acquire the cue hierarchy in English through exposure. They recognize that while pitch is informative, it is subordinate to pause and pre-boundary lengthening when making boundary decisions. Their estimated pitch weight (42.24%) was higher compared to monolinguals (29.63%), reflecting transferred sensitivity. However, it remained below their own weights for pause (80.46%) and lengthening (61.78%), implying L2-driven down-weighting.

In sum, cross-unit transfer and down-weighting appear to operate jointly. This finding extends prior formulations of cross-unit transfer (Kim, 2020; Tremblay et al., 2018) in that L1 functional weights can be reallocated in the L2 and can cross prosodic levels, from lexical tone to phrase-boundary processing, refining some of the predictions of the Cue-Weighting Transfer Hypothesis.

## 4. Conclusions

In two auditory experiments, we examined Chinese–English learners’ and monolingual English speakers’ sensitivity of English prosodic boundaries and identified the locus of observed processing difficulties. Contrary to accounts attributing L2 deficits to lack of awareness or global processing limits, our findings suggest that transfer of L1 cue-weighting strategies is a driving mechanism that is multi-cue, holistic, and dynamic. Learners integrate the L1 hierarchy into the L2 and then partially reweight it in response to L2 diagnostics, including cross-unit effects from lexical tone to phrase-level processing.

In sum, the contribution of the present study is two-fold. First, by testing boundary types and isolating individual acoustic cues, we have identified when and how transfer impairs boundary perception, refining the claims of the Cue-Weighting Transfer Hypothesis. Second, we have demonstrated that reweighting, rather than category absence, explains selective deficits, yielding potential implications for instruction that target cue calibration rather than generic prosody training. Future work should further explore these findings using other methods such as EEG to track neurocognitive indices of cue reweighting, to test the durability of training-induced shifts, and to model individual differences in L1-driven sensitivity. These endeavors would further develop our understanding of L1-to-L2 transfer and inform pedagogies that facilitate acquisition of L2 prosody.

## Figures and Tables

**Table 1:** Sensitivity to prosodic boundaries (*d*’) in Experiment 1.

	English Monolinguals	Chinese–English Learners
*d*’ no boundary	3.16 ± 2.05	2.67 ± 1.73
*d*’phrase boundary	3.96 ± 2.16	1.90 ± 0.81
*d*’sentence boundary	4.09 ± 2.24	3.92 ± 1.70

**Table 2:** Decision criteria (*C*) for prosodic boundaries in Experiment 1.

	English Monolinguals	Chinese–English Learners
*C* no boundary	0.29 ± 0.68	0.50 ± 0.56
*C* phrase boundary	0.31 ± 1.02	0.35 ± 0.29
*C* sentence boundary	−0.07 ± 0.84	−0.51 ± 0.99

**Table 3:** Identification rates (%) under cue conditions in Experiment 2.

	English Monolinguals	Chinese–English Learners
Baseline	95.06 ± 11.15	95.98 ± 4.79
Pause	79.94 ± 16.87	80.46 ± 14.13
Lengthening	82.41 ± 19.25	61.78 ± 18.97
Pitch	29.63 ± 12.52	42.24 ± 17.39

## Data Availability

The original data presented in the study are openly available at https://osf.io/xewq5/?view_only=6aef99f137fb4b4db9568287f993ab8a, accessed on 12 September 2025.

## Notes

1 We would like to thank Bei Wang for her valuable advice on the experimental design.

2 We thank an anonymous reviewer for bringing to our attention a limitation in our experimental design. While the stimulus set primarily consisted of compound words (e.g., jellybean, accounting for 70% of the materials), it also included some noun phrases (e.g., chocolate yogurt). According to the prosodic hierarchy, these two structures belong to different levels. A compound like jellybean typically forms a single prosodic word, whereas a noun phrase like chocolate yogurt constitutes a higher-level phonological phrase (i.e., intermediate phrase, see Selkirk, 2005). This structural distinction is accompanied by a significant difference in their canonical stress patterns. The compounds in our study typically had initial stress (e.g., JELLYbean), while the noun phrases had final stress (e.g., chocolate YOgurt). To ensure that these phonetic differences did not lead to different intonational contours, we conducted a post-hoc analysis. The findings revealed that this variability in stimulus type did not have a significant effect on our key results (specifically, at the ‘no-boundary’ and ‘phrase-boundary’ levels): Paired-samples *t*-tests comparing the two stimulus types at these levels showed no significant differences (all *p*s > 0.05) among both groups of participants.
